# Cobweb in Hemorrhage from Extracting Teeth

**Published:** 1872-02

**Authors:** 


*Cobweb in Hemorrhage from Extracting Teeth.*—Dr. Geo. S. Staples, of Corinth, Miss., writes as follows :

Editor *American Journal of Dental Science.* Your ever welcome Journal has just been received, and in its articles, I find one from Dr. Cutler on "Hemorrhage from Extraction." Some two or three years since I noticed—in same Journal—cobwebs recommended for arresting hemorrhage.

Since that time I have had occasion to use it three times and with the happiest results.

I will give you a brief account of the most obstinate case, if you think it worth the space you can publish it.

Mrs. H., aged about thirty-five years, called on me for the extraction of the left superior molar tooth,—this was about noon on Friday.

She experienced no trouble until about 3 o'clock on Sunday morning when the hemorrhage commenced. About 11 o'clock I was sent for in haste. I at once decided to try my experiment again. I found her spitting blood by the mouthful and considerably alarmed. I simply inserted a small pledget of the cobweb into each orifice from which the fangs of the tooth came, and in less than a minute the bleeding had ceased entirely.

I had intended relating my experience before so that others might try it, but up to the present time have neglected it."

